# Plant-Based Burgers Commercialized in the Spanish Market: Ingredients and Nutritional Assessment Based on Their Labels

**DOI:** 10.3390/foods14193286

**Published:** 2025-09-23

**Authors:** Iciar Astiasaran, Sheila Flores, Itziar Ariz-Hernandez, Diana Ansorena

**Affiliations:** 1 Department of Nutrition, Food Science and Physiology, Faculty of Pharmacy and Nutrition, University of Navarra, Irunlarrea 1, 31008 Pamplona, Spain; iastiasa@unav.es (I.A.); sflorestorr@alumni.unav.es (S.F.); iarizh@unav.es (I.A.-H.); 2 Center for Nutrition Research, Faculty of Pharmacy and Nutrition, University of Navarra, Irunlarrea 1, 31008 Pamplona, Spain; 3 IdiSNA, Navarra Institute for Health Research, 31002 Pamplona, Spain; 4 Instituto de Nutrición y Salud, Universidad de Navarra, 31009 Pamplona, Spain

**Keywords:** claims, formulations, labels, meat analogs, PBMAs

## Abstract

Plant-based burgers (covering 29 products from 18 different brands) commercialized on the Spanish market during the first semester of 2025 were analyzed based on the information provided on their labels. Most of the products (28) had between 11 and 26 ingredients, with a median of 16.0 (mean of 18.5). One product included 42 ingredients. Soy was the main source of protein (72% of the products), and olive and sunflower oils were the main fat sources (54 and 51% of the products, respectively). The median protein content was 11.0% (mean of 13.0%), with 80% of the burgers falling within the range of 5–16%. The median fat content was 9.7% (mean of 10.0%), with 80% of the products ranging from 5.8% to 13.3%. The proportion of carbohydrates exceeded 10% in 83% of the products, and the fiber content was higher than 2.9% in most of the products (75%). The median salt content was 1.2% (mean of 1.2%), and a low percentage of products used additives in their formulations. These results show that decreasing the number of ingredients and the amount of fat and salt remain challenges that need to be addressed in these types of products.

## 1. Introduction

Plant-based diets are a growing trend worldwide, due to environmental, ethical, and health reasons. Among the different choices of animal protein alternatives used for formulating animal food analogs, vegetal proteins are the best accepted by consumers [1]. This tendency is influenced by factors such as cultural acceptance, production costs, technological advancements, and their superior sensory properties compared to other alternatives [2]. Moreover, this option ensures the continuation of agricultural activities that are of significant importance to the economy and the development of communities [1].

In Europe, retail sales volumes of plant-based foods have been increasing in recent years in France, Germany, Italy, and Spain, largely driven by the growth of more affordable private-label products [3]. In the Spanish market, for example, 22% of the population bought plant-based meat analogs (PBMAs) at least once in 2024 [3].

PBMAs are specifically designed to mimic the sensory characteristics and nutritional aspects of meat [4]. Attention to the design of ingredient systems for the optimization of flavor, texture, binding, color, and nutrition is necessary for the development of plant-based meat alternatives with desirable consumer attributes [5]. Currently, the food industry incorporates specific ingredients and technologies in their production process. While soy protein initially dominated the market, the industry has since diversified, incorporating a wide range of plant-based protein sources such as pea, wheat, potato, mung bean, oat, and rice [6]. In addition, flavoring agents, fats, and additives are introduced to enhance the sensory appeal, with binding agents, texturizers, flavors, and coloring agents playing crucial roles in product enhancement [7].

Given the presence of additives such as stabilizers, colorants, and preservatives, PBMAs are considered ultra-processed foods (UPFs) and their nutritional quality, as well as their perception by consumers, can be compromised [8].

The nutritional composition of these products varies depending on the type of formulated product (sausages, burgers, nuggets, meatballs, etc.), protein sources and processing methods used [4,9]. The processing to which ultra-processed plant-based analogs are subjected to acquire the characteristics of animal-derived products might result in an opposite effect, producing unhealthy ultra-processed foods [10]. PBMAs frequently contain anti-nutrients, have less protein, iron, and vitamin B12, are lower in protein quality, and also have higher amounts of sodium [11]. Shireen and Wright [12], analyzing the nutritional quality of plant-based meat analogs versus traditional meat products, concluded that it cannot be assumed that the former are healthier than the latter. Moreover, evidence regarding the long-term health effects of consuming PBMAs, especially new products, is lacking [13]. After a thorough study on the plant-based meat analogs commercialized on the Italian market (FLIP project), Cutroneo et al. [14] concluded that there exist a need to improve the formulation of meat analogs in terms of the number of added ingredients and processing.

The aim of this work was to provide a concise overview of the most commonly used ingredients and the nutritional profiles of commercially available plant-based burgers on the Spanish market today (first semester 2025). All the information was collected from the labels.

## 2. Materials and Methods

Commercial plant-based burgers were identified (from January to March 2025) through the websites of different brands that commercialize their products on the Spanish market. Table 1 shows the number of products, corresponding to 18 different brands. Twenty-nine different products were selected and information from the list of ingredients used in their formulation and nutritional data were collected from their labels.

Data about the number of ingredients and amounts of energy (kcal), protein (g), fat (g), saturated fat (g), carbohydrates (g), sugars (g), fiber (g), and salt (g) per 100 g of product were first evaluated for normality using the Shapiro–Wilk test. This test was chosen due to its suitability for small sample sizes. All the parameters showed a normal distribution (*p* > 0.05), except the number of ingredients (*p* = 0.00003) and the saturated fat (*p* = 0.0001). Data were categorized into quartiles. The number of products per quartile (absolute frequencies) and their percentage over the total amount of products (relative percentages) were calculated and represented in the figures. Also, the median and mean values for each parameter were calculated and reported for discussion purposes. The standard composition of a beef meat burger [15] was used to discuss the nutritional differences from traditional burgers.

## 3. Results and Discussion

### 3.1. Ingredients

**Figure 1:**
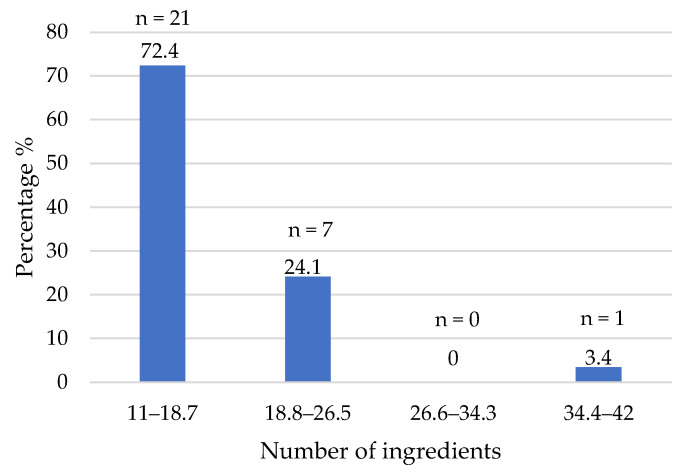
Ingredients used in the formulations of the plant-based burgers (number and percentage of products per quartile).

The total list of ingredients ranged from 11 to 42 (Figure 1), and most of them (28) included between 11 and 26 ingredients, with a median of 16.0 and a mean of 18.5 ingredients per product.

One of the plant-based burgers showed a total of 42 ingredients. A mean of 13.4 ingredients was found in plant-based burgers (*n* = 25) commercialized in Barcelona (Spain), with a range of 9–22 ingredients [16]. These data show that there has not been a decrease in the number of ingredients used in the formulation of these types of products in recent years. After the analysis of the consumer perception of plant-based burgers, it was concluded that the length of the ingredient list is more important for consumer perceptions of clean labels than the presence of chemical additives per se [17].

Table 2 shows the type of ingredients and the number of products containing them. Every product included plant protein sources, vegetable oils, vegetables, salt, and spices.

The type of protein source used in the plant-based burgers is summarized in Table 3.

The main protein source was soy, used in 72% of the burgers, followed by wheat in 48% of burgers, pea in 28%, and lentils in 7%. Soy protein is considered an ideal ingredient for producing plant-based meat alternatives due to its favorable processing characteristics, balanced nutritional profile, and relatively low cost [18].

To replicate the juiciness, tenderness, and flavor of traditional meat, vegetable oils play a key role [7]. The most commonly used oils (Table 4) were sunflower oil (in 52% of the products) and extra virgin olive oil (41%). Moreover, olive oil (mix of refined and virgin oil) and virgin olive oil were also used in another 10.3% of the products. Other oils, including coconut, canola, palm, rapeseed, and shea, were used in only a small number of products (one or two each). Vegetables (such as carrot, tomato, onion, zucchini, and beetroot) and spices (including garlic, pepper, parsley, oregano, curry, and cocoa powder) were also included in all formulations, contributing to the potential presence of bioactive compounds in the final products. Moreover, vegetables, such as beetroot, and cocoa powder were likely used to enhance the color of the final product, minimizing the need for added colorants, which were natural and only used in 27% of the burgers. Salt (sodium chloride) was a consistent ingredient across all products (28 products included salt), essential not only for flavor, but also for preservation. Sea salt was used in twelve products, and Himalayan pink salt in seven. Potassium salt was also used in one of the products.

Texturizing additives, which aim to enhance the interaction of water with the lipid and protein parts of the matrix, were used in 27 products. Methylcellulose appeared in eight products and several gums (agar-agar, gellan, and xanthan) in five products. It has been pointed out that methylcellulose could be a good option to use as a sole binding agent in soybean burgers [19]. Also, the formulations containing with tofu (10 products) included magnesium chloride (nigari) as a texturizing agent. It is worth noting that 17 products included gluten, added as seitan (9 products) or directly as gluten or wheat. Gluten improves the water-holding retention in plant-based meat analogs, and its use is directly related to the final texture of the product [20,21]. Also, 76% of the products included some type of carbohydrate source, such as starches from corn, potato or tapioca in the formulation, and 62% of products included yeast or fermented products. Other ingredients such as fibers, sugars, seaweeds, fruits, or seeds were found only in a limited number of products. The use of preservatives (sorbates, metabisulfites, acids) or antioxidants (ascorbic acid) were found in 24% of the products. Some products (30–40%) contained natural aromas and colorants. Iron and vitamin B12 were found in two types of burgers from the same brand. Rizzolo-Brime et al. [22] analyzed the composition of plant-based meat alternatives and found that these nutrients were added in 11 out of 148 products.

### 3.2. Nutritional Aspects

**Figure 2:**
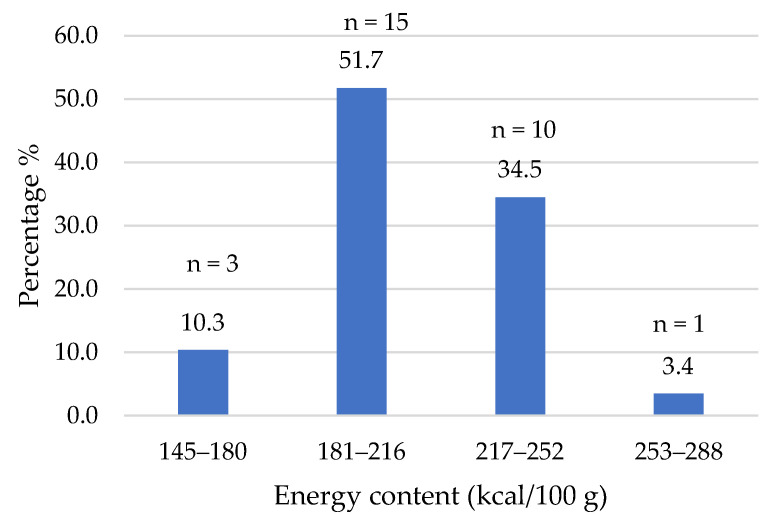
Energy value (kcal/100 g) of plant-based burgers (number and percentage of products per quartile).

**Figure 3:**
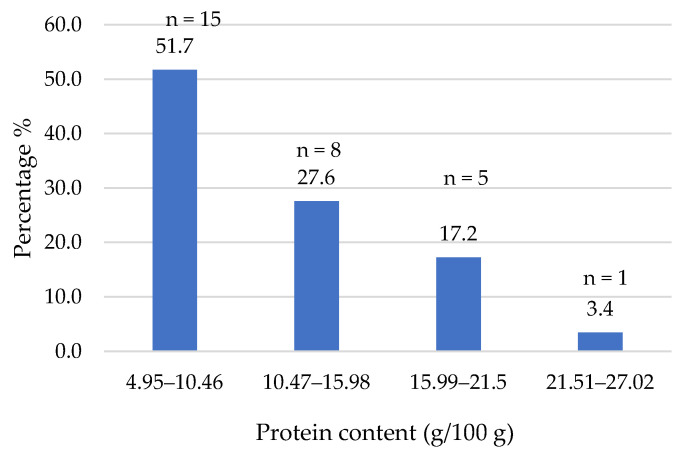
Protein content (g/100 g) of plant-based burgers (number and percentage of products per quartile).

**Figure 4:**
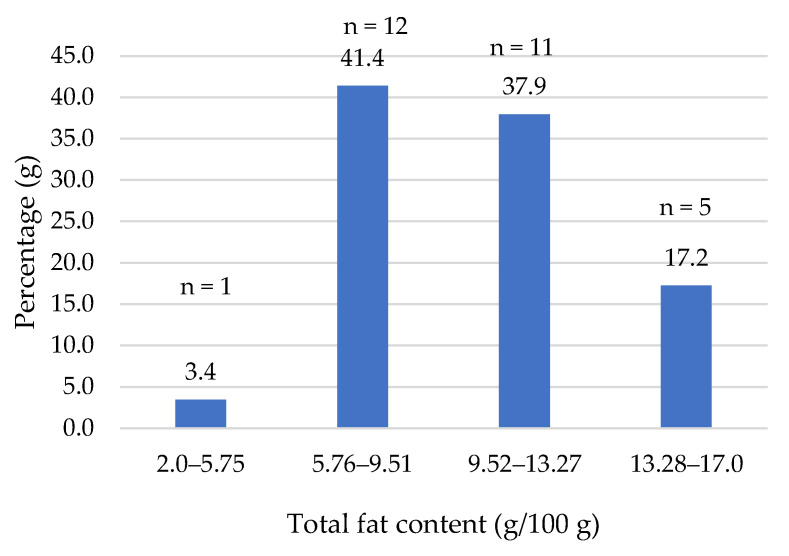
Total fat content (g/100 g) of plant-based burgers (number and percentage of products per quartile).

**Figure 5:**
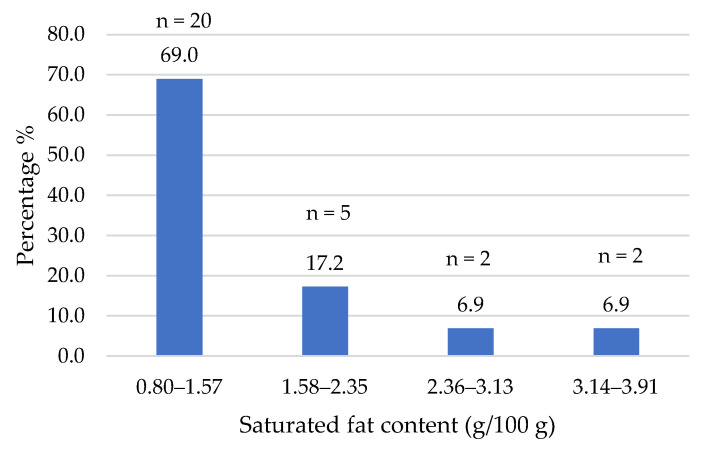
Saturated fat content (g/100 g) of plant-based burgers (number and percentage of products per quartile).

**Figure 6:**
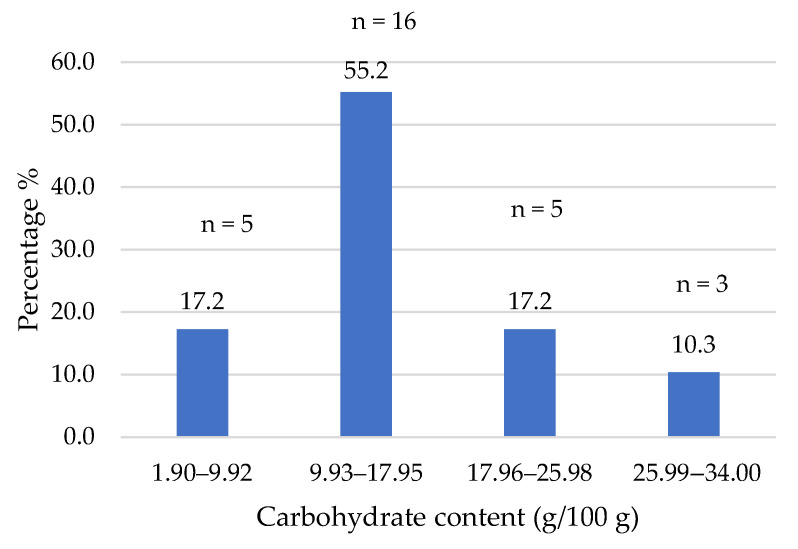
Carbohydrate content (g/100 g) of plant-based burgers (number and percentage of products per quartile).

**Figure 7:**
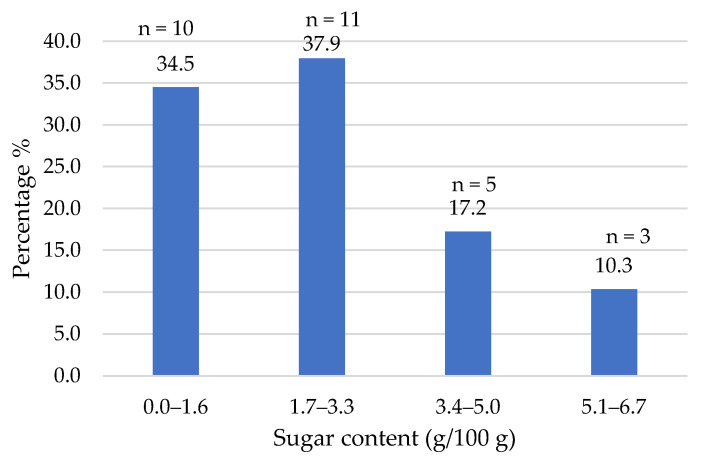
Sugar content (g/100 g) of plant-based burgers (number and percentage of products per quartile).

**Figure 8:**
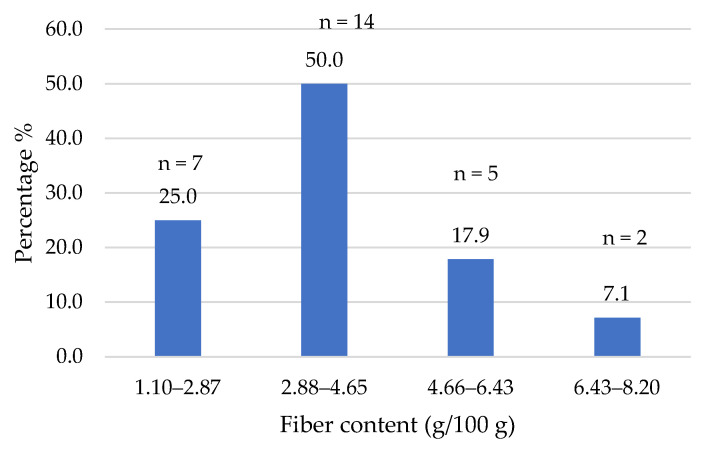
Fiber content (g/100 g) of plant-based burgers (number and percentage of products per quartile).

**Figure 9:**
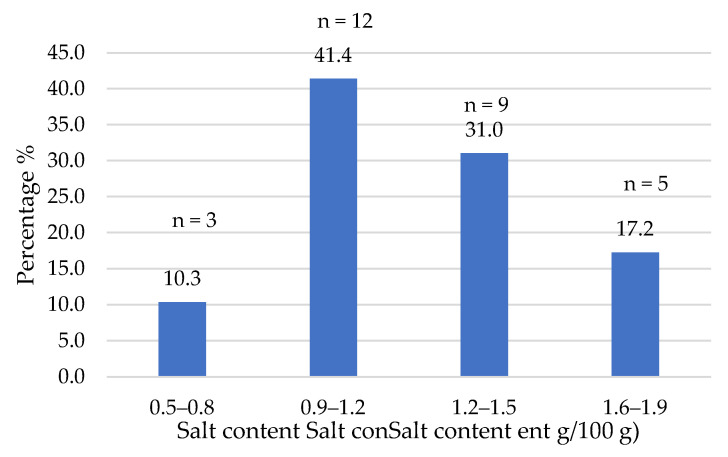
Salt content (g/100 g) of plant-based burgers (number and percentage of products per quartile).

The composition data of the commercial products studied were obtained from the nutritional information provided on their labels. Figure 2, Figure 3, Figure 4, Figure 5, Figure 6, Figure 7, Figure 8 and Figure 9 show the results per 100 g of product: energy value, total fat, saturated fat, carbohydrates, sugars, fiber, protein, and salt. The values were grouped into quartiles, determining the number and percentage of products falling within each range. For comparison purposes, the composition of a standard beef burger [15] was used (Table 5). It is important to note that all values corresponded to the raw products (as reported on the labels), and thus do not reflect the potential modifications which may occur during cooking, especially as a consequence of water losses. In addition, it should be noted that losses due to cooking in PBMAs are lower than in traditional meat products [9,23].

Regarding the energy content (Figure 2), more than 50% of the products (*n* = 15) provided between 181 and 216 kcal per 100 g.

These values are consistent with those previously reported [4]. Only 10% of plant-based burgers exhibited energy values similar to those of a standard beef burger (166 kcal/100 g). The remaining products showed higher values, with one product reaching up to 288 kcal/100 g.

In terms of the protein content (Figure 3), it is noteworthy that the majority of plant-based burgers (at least 80%) showed a protein content within the range 5–16%. These values are quite similar to those obtained after laboratory analysis of seven different plant-based burger brands commercialized on the Brazilian market (5.25–13.55%) [24]. Slightly higher values (8.9–22.0%) were found on the Spanish market when analyzing the labels of plant-based burgers (*n* = 20) [22]. Cutroneo et al. [14], analyzing different types of plant-based meat analogs on the Italian market, observed that, in the case of burgers, the amount of protein was lower than in their meat counterparts (12 and 17%, respectively). In our study, only one product was included in the fourth quartile, showing 27% protein. The median value for the 29 products was 11.0% and the mean value was 13.0% protein, which was similar to the mean values found on the Australian market [25], the Swedish market [26] and the European market [27]. Although traditional meat burgers can also show a certain variability, in general, their amount of protein is higher (19.4%, Table 5). These results indicate that most of the formulations fail to provide optimal protein levels to be considered true alternatives to traditional beef burgers. However, it is true that they are a good source of protein and comply with the criteria established in Regulation 1924/2006 [28] for bearing protein-related claims. Twenty products could include the claim “high protein” and seven products could bear the claim “source of protein”. It is worth noting that only 11 products actually make use of this type of claim. Moreover, it should be pointed out that in half of the products, a mix of cereals and legumes was used, which could improve the quality of the supplied protein, combining the amino acids provided by these two food groups.

Fat is the macronutrient that provides the highest energy value. As shown in Figure 4, most of the products (80%) had a fat content ranging between 8.2 and 14.2%, with a median of 9.7% (mean of 10.0%), suggesting that their lipid supply is quite similar to that of a standard beef burger (10%, Table 5).

However, notable differences were found in the amount of saturated fatty acids and, as a consequence, in the fatty acid profiles of these lipids (Figure 5).

As was expected, the supply of saturated fatty acids was low, in the range of 0.8–1.57% in 69% of the burgers. Only five products showed saturated fatty acid amounts higher than 2%, with a maximum of 3.9% in one of the burgers. This low supply of saturated fatty acids, with a mean of 1.3% (median of 1.3%), is clearly a nutritional advantage in contrast with traditional beef burgers, which usually show higher amounts of saturated fatty acids (4.4%, Table 5). Previous data obtained for plant-based burgers commercialized on the Spanish market some years ago gave rise to similar values (1.9% [16], 1.25% [22]). Lima et al. [29], in a study on plant-based meat alternatives in the Brazilian market, found much higher values of saturated fatty acids (5.8%) in burger samples. According to Regulation (EC) No. 1924/2006, a nutritional claim of “low saturated fat” may be used when the total saturated fatty acid content is below 1.5% and less than 10% of the product’s energy value is derived from the sum of these fatty acids and trans fatty acids [28]; 57% of products met both criteria, suggesting that this claim could be valid for labeling. However, only three of the products showed this claim on their labels. The use of vegetable oils in the formulations of plant-based meat analogs instead of animal fats also leads to the absence of cholesterol in this type of food. Oh et al. [30] emphasized the higher content of fat, saturated fatty acids and cholesterol of beef products when compared with plant-based meat analogs on the Korean market.

Unlike meat, which contains negligible amounts of carbohydrates, plant-based ingredients including texturized proteins, starches, vegetables, fruits, sugars, and seeds, which contribute to their carbohydrate content. In this study, 83% of plant-based burgers contained more than 10% carbohydrates, with 10% of products having between 25% and 34% (Figure 6), and a median of 14.2% (mean of 15.1%). Other studies reported a wide range of values for this parameter (1.4% to 59.7%), with a mean value of 12.1% for similar products [29]. This macronutrient significantly contributes to the energy values of the burgers.

Most carbohydrates present in the analyzed products were complex, particularly starches, whereas the sugar content remained relatively low (maximum 6.7%) (Figure 7). These findings align with the ingredients used, as only five products included added sugars or syrups. According to Regulation 1924/2006, most plant-based burgers could be classified as “low in sugars,” since they contain less than 2.5 g of sugar per 100 g [28]. In fact, three products showed the claim “without added sugars”.

Fiber content is another important nutritional aspect of PBMAs in contrast to traditional meat products. Most products (75%) had a fiber content above 2.9% (Figure 8). The mean value for the analyzed burgers (28 in this case, as one of them did not show the fiber content on the label) was 3.7% (median of 3.7%), similar to the results found in previous works (3.5% by Costa-Catala et al. [16]; 3.8% by Bryngelsson et al. [26]; 4.0% by Rizzolo-Brime et al. [22] and Filip et al. [27]). A higher mean value was reported in Brazilian burgers (5.2%), with values ranging from 0 to 10.4% [29]. These amounts are considered significant, given that foods with ≥3% fiber can be labeled as a “source of fiber,” and those with ≥6% as “high in fiber” [28]. However, only 6 out of the 18 products with fiber content above 3% showed the claim “source of fiber”, and none of them showed the claim “high in fiber”.

In a review that compared plant-based meat analogs with their meat counterparts, it was concluded that they typically contain higher amounts of salt [31]. In our study, over 70% of the products contained between 0.9 g and 1.5 g of salt per 100 g (Figure 9), whereas 17% contained between 1.6 g and 1.9 g. The median of all analyzed burgers was 1.2% (mean of 1.2%) salt, which is lower than the mean value of a standard beef burger (730 mg sodium, Table 5). Filip et al. [27] also found salt amounts in commercial meat burgers in the range of 0.3–2.2%. However, it is clear that the consumption of plant-based burgers does not necessarily result in lower salt intake. In plant-based foods, salt is primarily added to enhance flavor, unlike in meat products, where it also serves a preservative role due to the need for higher microbiological stability. Costa-Catala et al. [16] reported that salt was present in more than 90% of 100 meat analogs (burgers, meatballs, sausages, and nuggets) analyzed in that work, making it the most frequently used ingredient.

## 4. Conclusions

This work aims to show the current characteristics of plant-based burgers commercialized on the Spanish market in 2025 through an analysis of their labels. The results show that, in general, they are formulated with a large number of ingredients (11–26), always including plant protein sources, vegetable oils, vegetables, salt and spices. They supply a significant amount of protein (above 12% of the total energy value of the products), although, in general, this is lower than that for traditional beef burgers. They contain high levels of total fat, carbohydrates, and salt, but compared to traditional beef burgers, their saturated fatty acid content is much lower. They can also be considered a good source of fiber. These data show that the composition of commercialized plant-based burgers has not improved in recent years from a nutritional point of view. We recommend further research and innovation in plant-based meat analogs in order to offer consumers products with healthier nutritional profiles, especially with decreased fat and salt contents.

## Figures and Tables

**Table 1:** Brands of plant-based burgers identified and number of products analyzed per brand.

Brand	Number of Products
Soria natural	5
El Corte Inglés Special Line	3
Gerblé	2
Hacendado	2
Heura	2
NaturSoy	2
Vegetalia	2
Beyond meat	1
Di que sí	1
Eroski	1
Garden gourmet	1
KoRo	1
Obrador Sorribas	1
Schar	1
Toki	1
Valsoia	1
Vemondo	1
Veritas	1

**Table 2:** Summary of the ingredients used in the plant-based burgers by category and the number of products containing them.

Category	Number of Products	Ingredient Description
Plant Protein Source	29	Soy, pea, seitan (wheat gluten), lentils
Vegetable Oil	29	Rapeseed, coconut, extra virgin olive oil, sunflower, shea fat, canola
Vegetables	29	Vegetable concentrate, carrot, onion, beetroot, artichoke, mushrooms, zucchini, spinach, eggplant, red bell pepper, hibiscus, tomato
Salts	29	Sodium chloride, sea salt, Himalayan pink salt, potassium salt
Spices	29	Ginger, garlic, pepper, nutritional yeast, aromatic herbs, onion powder, parsley, oregano, curry, sweet paprika, turmeric
Texturizing Agents	27	Methylcellulose, agar-agar, gellan gum, xanthan gum, konjac glucomannan, calcium chloride, nigari (magnesium chloride), gluten
Carbohydrate Source	22	Wheat, corn, corn starch, barley, whole wheat, tapioca starch, potato starch, rice, oats, potato starch, fermented rice, brown rice, polenta, oat flakes, hydrated wheat bulgur, breadcrumbs, cooked white quinoa, buckwheat flour
Yeast/Fermented Products	18	Yeast, soy sauce (shoyu-koji ferment), vinegar, fermented rice
Aromas	12	Natural aroma, smoke aroma
Fiber	8	Dietary vegetable fiber, soy fiber, vegetable fiber (bamboo, gluten-free oats, psyllium)
Colorants	8	Beetroot extract, curcuma, cocoa
Sugars	5	Malt extract, sugar, rice syrup, caramelized sugar syrup, dextrose, cane sugar
Algae	5	Kombu seaweed, sea spaghetti seaweed
Preservatives/Antioxidants	7	Potassium sorbate, sodium metabisulfite, citric acid, lactic acid, potassium lactate, ascorbic acid
Fruits	2	Rehydrated sultanas, dates, apple concentrate, concentrated pomegranate juice
Seeds and Nuts	2	Toasted sesame, pine nuts, almonds
Vitamins	2	Vitamin B12
Minerals	2	Iron

**Table 3:** Frequency and distribution of protein source ingredients used in the evaluated plant-based burger formulations.

Ingredient	Number of Products	% of Products
Soy	21	72.4
Wheat	14	48.3
Pea	8	27.6
Lentils	2	6.9

**Table 4:** Frequency and distribution of vegetable oils used in the evaluated plant-based burger formulations.

Ingredient	Number of Products	% of Products
Sunflower	15	51.7
Extra virgin olive oil	12	41.4
Olive oil	3	10.3
Coconut	2	6.9
Canola	2	6.9
Palm	2	6.9
Virgin olive oil	1	3.4
Rapeseed	1	3.4
Shea fat	1	3.4

**Table 5:** Nutritional composition per 100 g of a standard beef burger [15].

Energy and Nutrients	Value Per 100 g of Beef Burger
Energy (kcal)	166
Protein (g)	19.4
Lipids (g)	9.6
Saturated fats (g)	4.37
Monounsaturated fats (g)	4.92
Carbohydrates (g)	0.6
Water (g)	67.6
Sodium (mg)	730
Iron (mg)	2.2
Zinc (mg)	5.1

## Data Availability

The original contributions presented in this study are included in the article. Further inquiries can be directed to the corresponding author.
